# Design of an In Vitro Model for Epithelial-to-Mesenchymal Transition in Gastric Cancer

**DOI:** 10.1007/s10528-024-10668-x

**Published:** 2024-03-20

**Authors:** Yuanhui Zhang, Ling Bi, Quanyao Li, Liqiu Yao, Xiao Wang, Hui Liu, Jun Shi

**Affiliations:** 1 Department of Oncology, Yueyang Hospital of Integrated Traditional Chinese and Western Medicine, Shanghai University of Traditional Chinese Medicine, Shanghai, 200437 China; 2 Department of Traditional Chinese Medicine, Shanghai Fourth People’s Hospital Affiliated to Tongji University of Medicine, Shanghai, 200434 China

**Keywords:** Epithelial-to-mesenchymal transition, Gastric cancer, IL-8, In vitro model, Metastasis

## Abstract

Epithelial-to-mesenchymal transition (EMT) is a developmental program that plays a vital role in gastric cancer, including aspects of tumor progression, the metastatic process, and resistance to treatment. Here, we have designed an in vitro model that mimics the features of EMT as observed in gastric cancer. The results showed that both migration and invasion were enhanced in gastric cancer cells with Brachyury overexpression. Additionally, the expression of IL-8 increased, while IL-8RA and IL-8RB levels significantly decreased in the in vitro model. Overall, the in vitro model offers an opportunity to study these phenomena relevant to EMT as they may occur in vivo in gastric cancer, as well as potential drug interactions that could interfere with these processes.

## Introduction

Gastric cancer (GC) is not only one of the most common forms of cancer worldwide but also exhibits a high mortality rate. Late diagnosis often leads to deaths primarily due to distant metastasis among GC cases (Abbaszadegan et al. 2020). A better understanding of the mechanisms that underlie GC initiation and progression is essential for improving clinical outcomes.

The epithelial-to-mesenchymal transition (EMT) is a dynamic process and the cells trans-differentiate into two or more somatic states (Raja et al. 2020), which occurs not only during embryonic development but also during tumor progression. Increasing evidence demonstrates that EMT plays a vital role in the progression of many cancer types including gastric cancer, with involvement in tumor proliferation, progression, the metastatic process, and resistance to therapy (Brabletz et al. 2018). Epithelial cells lose their cell polarity and cell–cell adhesion and simultaneously gain migratory and invasive properties to become mesenchymal stem cells (Brabletz 2012). The molecular trait of EMT is the downregulation of epithelial marker E-cadherin and upregulation of mesenchymal marker N-cadherin, vimentin, and so on. E-cadherin, encoded by the *CDH1* gene, is switched to N-cadherin, encoded by the *CDH2* gene is an important event in EMT (Huang et al. 2015). This change in balance causes the transitional Loss of cells the original epithelial cell phenotype, thus acquiring a mesenchymal phenotype.

The T-box transcription factor Brachyury is involved in the development of vertebrates, including differentiation of the posterior mesoderm and axial development (Naiche et al. 2005). Palena et al. first described the role of Brachyury in human tumorigenesis in 2007, showing that Brachury mRNA is present in a variety of human tumors, including stomach, small intestine, and kidney, among others (Palena et al. 2007). Previous research has confirmed that Brachyury protein is involved in the process of carcinogenesis and progression of chordoma and several epithelial carcinomas in various studies (Chen et al. 2020). Recent studies have shown that high levels of Brachyury expression were related to metastasis and poor prognosis in prostate cancer and hepatocellular carcinoma and gastrointestinal stromal tumors (Du et al. 2014; Pinto et al. 2014, 2016). In chordoma cells, Brachyury blocked the effect of FGF on EMT by upregulating E-cadherin expression and downregulating snail expression (Hu et al. 2014). This implies that Brachyury-expressing cells could be used to mimic many features of EMT in cancer cells.

In this report, we propose that, compared to existing EMT models, in vitro models using this cancer cell line may offer a more accurate in vivo representation of cancer.

## Materials and Methods

### Cell Lines and Cultures

Human gastric cell lines, MGC803, BGC823, and SGC7901, were obtained from ATCC. All cell lines were cultured according to the recommendations provided by ATCC. The MGC803 and SGC7901 cell lines were cultured in DMEM medium, while the BGC823 cell line was cultured in RPMI-1640 medium. Both media were supplemented with 10% (v/v) fetal bovine serum (FBS; Invitrogen, USA) and 1% penicillin and streptomycin. Cells were maintained at 37 °C in an atmosphere of 5% CO_2_ and 95% air.

### Vector and Transfection

Lentiviral plasmids expressing human Brachyury gene were purchased from Shanghai Ruisai Biotechnology Co., Ltd. Brachyury cDNA was cloned into the restriction site of the lentiviral plasmid pLenti6.3-IRES2-EGFP/V5, which contains multiple cloning sites, to generate pLenti-Brachyury-EGFP for Brachyury gene overexpression. The Brachyury-expressing lentivirus was produced using the following protocol. Overexpression plasmid pLenti-Brachyury-EGFP and packaging plasmid pLP1-Gag-pol, pLP-VSVG, and pLP2-Rev transfected into H293T cells. The transfection was facilitated by using the reagent POLO Deliver 3000, acquired from Shanghai Ruisai Biotechnology Co., Ltd. After 48 h of infection in H293T cells, the viral supernatant was collected and centrifuged at 4 °C for 15 min at 3000 rpm. The centrifuged viral supernatant was filtered through a 0.45 μm membrane and the filtered solution was stored at − 80 °C.

The Brachyury lentivirus was used to infect the gastric cell lines. Details are as follows: (1) The cell suspension was inoculated in a 6-well cell culture plate and cultured overnight in a 5% CO_2_ incubator at 37 °C. (2) An appropriate volume of both target and control viruses was added to the cells in 2-well plates, based on their MOI values (MGC803, MOI = 50; BGC823, MOI = 20; SGC7901, MOI = 20). The expression of the reporter gene, GFP, was monitored 48 h post-infection and documented photographically. After harvesting the cells, we performed Western blot, real-time PCR, immunofluorescence, and additional experiments.

### Western Blot Analysis

Cells were lysed using RIPA lysis buffer (Santa Cruz Biotechnology Inc.). Following the normalization of protein concentration, the samples were separated using 12% SDS-PAGE electrophoresis and then transferred to nitrocellulose membranes (Bio Rad, Hercules, USA). The appropriate primary antibody was incubated overnight at 4 °C. Subsequently, we incubated the membrane with a secondary antibody coupled to horseradish peroxidase and detected it using an enhanced chemiluminescence substrate (Western Lightning Plus-ECL, PerkinElmer Inc, USA). All membranes were stripped and then re-probed with anti-GAPDH antibodies to verify the equal loading. All washing and incubation steps were conducted under continuous stirring. The protocol for using the primary antibodies is as follows: E-cadherin (Cell Signaling Technology, Cat.#:14472), N-cadherin(Cell Signaling Technology, Cat.#:14215), Vimentin (Cell Signaling Technology, Cat.#:5741), Fibronectin (Abcam, Cat.#:ab2413), Slug(Cell Signaling Technology, Cat.#:9585), Snail (Abcam, Cat.#:ab180714), and Brachyury (Abcam, Cat.#:ab20680). The rabbit anti-mouse secondary antibody (Cat.#:315-035-003) and goat anti-rabbit secondary antibody (Cat.#:111-035-008) were obtained from Jackson ImmunoResearch Inc (West Grove, PA, USA).

### RNA Extraction and Quantitative Real-Time PCR

Total RNA was extracted using TRIzol reagent (Invitrogen, USA), following the manufacturer’s guidelines. The concentration and purity of the total RNA were evaluated using a Nanodrop Spectrophotometer (Thermo Fisher Scientific, USA). The total RNA was converted to cDNA using First Strand cDNA Synthesis protocols (New England Biolabs, Ipswich, MA, USA), as per the manufacturer’s guidelines. For mRNA expression analysis, reverse transcription was carried out using SYBR-GreenER qPCR SuperMix for iCycler (Life Technologies, Grand Island, NY, USA), in accordance with the manufacturer’s protocol. Three independent experiments were conducted. Quantitative real-time PCR analyses were run in triplicate using the GeneAmp PCR System 9700 (Applied Biosystems, USA). Actin served as the endogenous control in all reactions. GAPDH was utilized as the reference gene for internal control in all reactions. Three independent experiments were conducted. Complete primer sequences are detailed in Tables 1 and 2.

**Table 1:** Primers for polymerase chain reaction

Gene	Forward (5′ to 3′)	Reverse (5′ to 3′)
β-actin	GGCACTCTTCCAGCCTTCC	GAGCCGCCGATCCACAC
E-cadherin	AATGCCGCCATCGCTTAC	TCAGGCACCTGACCCTTGTA
N-cadherin	CCATCAAGCCTGTGGGAATC	CCACTGCCTTCATAGTCAAACAC
Vimentin	GTCCACTGAGTACCGGAGACA	CGAAGGTGACGAGCCATTT
Fibronectin	CGGTGGCTGTCAGTCAAAG	AAACCTCGGCTTCCTCCATAA
Snail	TAGCGAGTGGTTCTTCTGCG	TTAGGCTTCCGATTGGGGTC
Slug	TGAGGAATCTGGCTGCTGTG	CAGGAGAAAATGCCTTTGGACT

**Table 2:** Primers for polymerase chain reaction

Gene	Forward (5′ to 3′)	Reverse (5′ to 3′)
β-actin	TCCTTCCTGGGCATGGAGT	CAGGAGGAGCAATGATCTTGAT
IL-8	ACATACTCCAAACCTTTCCACC	AAAACTTCTCCACAACCCTCTG
IL-8RA	CCTTTTCCGCCAGGCTTAC	GCAGGACCAGGTTGTAGGG
IL-8RB	GCCTGTCTTACTTTTCCGAAGG	GCAGTGGCACGATGAAGC

#### In Vitro Cell Migration and Invasion Assay

Cell migration and invasion assays were conducted using Transwell chambers with 8 µm pores (Corning Incorporated, Corning, NY, USA). The Transwell chambers were pre-coated with Matrigel matrix for the invasion assay. Briefly, 5 × 10^4^ MGC803, SGC7901, and BGC823 cells, which were transfected either with Lenti-Brachyury or Lenti-EGFP, were seeded in the upper chamber containing RPMI-1640 medium. RPMI-1640 medium supplemented with 10% FBS was then added to the lower chamber. After 24 h of incubation at 37 °C in a humidified atmosphere containing 5% CO_2_, a cotton swab was used to remove cells from the surface of the upper compartment of the membrane. Cells that penetrated the pores and migrated to the lower side were fixed with paraformaldehyde and stained with 0.1% crystal violet. Cells were imaged and quantified using an inverted microscope (Olympus Corporation, Tokyo, Japan).

### Immunofluorescence

Immunofluorescence was performed as previously described (Wen et al. 2020). The cell lines MGC803, BGC823, and SGC7901 were cultured on glass slides, fixed with 4% paraformaldehyde for 15 min, and blocked with BSA at room temperature for 1 h. Subsequently, the cells were thrice washed with 0.1% Triton X-100/PBS. Next, the cells were incubated overnight with E-cadherin (Cat.#:14472), N-cadherin (Cell Signaling Technology, Cat.#:14215), and Vimentin (Cell Signaling Technology, Cat. #: 5741). The nuclei were re-stained with DAPI (Beyotime, China) for a duration of 5 min. Finally, the cells were mounted in a 50% glycerol/PBS solution. Imaging was conducted using a Carl Zeiss LSM 710 microscope.

### Flow Cytometry

Flow cytometric analysis was employed for the detection of IL-8RA and IL-8RB markers in gastric cancer samples. In summary, following Lenti-Brachyury transfection, a single-cell suspension comprising MGC803, BGC823, and SGC7901 gastric cancer cell lines was incubated with Anti-CXCR1 (IL-8RA) (Abcam, Cat.#:ab89251) and Anti-CXCR2 (IL-8RB) (Abcam, Cat.#:ab89254). Samples were then left in darkness at 4 °C for 30 min. After washing, the labeled cells were resuspended in PBS and subsequently analyzed using BD FACSCalibur (BD Biosciences, San Jose, CA, USA).

### Quantitation of IL-8 by Enzyme-Linked Immunosorbent Assay (ELISA)

The protein expression levels of IL-8 in the MGC803, BGC823, and SGC7901 cell lines were assessed using ELISA following the treatment with Lenti-Brachyury. After 48 h of exposure to Lenti-Brachyury, the cultured supernatants from the three gastric cell lines were collected. The levels of IL-8 were assessed using ELISA (R & D Systems, D8000C). The detection methods have been described previously (Kim et al. 2018). Briefly, we sealed each well for 2 h using a sealing buffer and then washed them with a wash buffer. Antibodies against IL-8 were added to the medium and incubated for 2 h. A substrate solution followed by a stop solution was added sequentially. The optical density of each well was measured within 30 min using a microplate reader (Thermo Scientific, Multiskan mk3).

### Statistical Analysis

All statistical analyses were conducted using SPSS 26.0 software (SPSS Inc.).The results are presented as the mean ± standard deviation, based on at least three independent experiments. Differences between groups were assessed using an unpaired, two-tailed Student’s t-test. A *p*-value of < 0.05 was considered to indicate statistical significance.

## Results

### Establishment of an EMT Model in Gastric Cancer Cell Lines

To establish a model of EMT, which is characterized by decreased levels of epithelial signature proteins like E-cadherin and increased levels of mesenchymal signature proteins like N-cadherin and Vimentin, it is crucial to first screen for ideal gastric cancer cell lines. For the screening process, cell lines should exhibit relatively high expression of E-Cadherin, a characteristic protein of epithelial background, and relatively low expression of Vimentin, a characteristic protein of the mesenchymal background. We utilized E-Cadherin and Vimentin as markers to represent epithelial and mesenchymal characteristics, respectively. These markers were used to assess the background expression in various gastric cancer cell lines and ultimately to identify the most suitable cell lines for the EMT model.

**Fig. 1:**
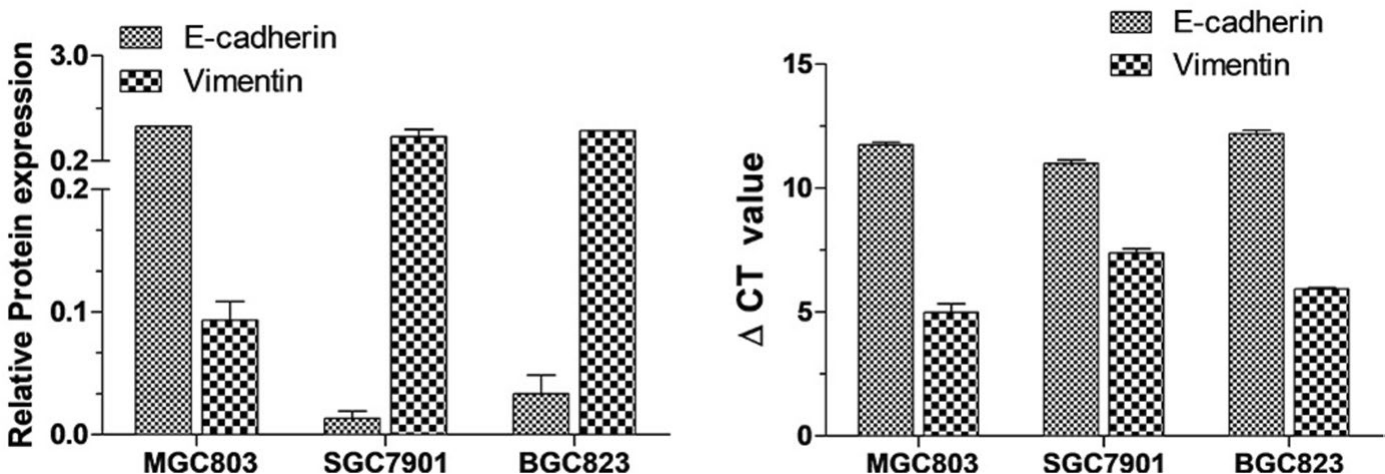
Protein and mRNA expression of E-Cadherin and vimentin in three types of gastric cancer cells. Gastric cancer cells were seeded at a density of 1 × 10^6^ cells per well in 6-well plates. Once cells reached 80% confluency, they were collected for Western blot and RT-PCR analyses. The experiment was conducted in triplicate and repeated three times

We conducted a search in the PubMed electronic database, aligned with the objectives of this study and based on the frequency of citations in EMT-related research. We selected three human gastric cancer cell lines according to their degree of differentiation: highly differentiated MGC803 cells, moderately differentiated SGC7901 cells, and poorly differentiated BGC823 cells. Western blot and qPCR methods were employed to assess the expression levels of E-Cadherin and Vimentin proteins and genes across these three cell lines. Among the three cell lines—MGC803, SGC7901, and BGC823—E-cadherin expression was the highest in MGC803 cells, followed by BGC823, and the lowest in SGC7901 cells. Conversely, Vimentin expression was higher in SGC7901 and BGC823 cells compared to MGC803 cells. SGC7901 and BGC823 cells all have low expression of E-cadherin protein and high expression of Vimentin, which is much more obvious on SGC7901 cells (Fig. 1).

Therefore, both SGC7901 and BGC823 cells are suitable for constructing an EMT model. Within this context, SGC7901 cells exhibited greater variability in the expression levels of the two target proteins, making them particularly suitable for model construction. SGC7901 cells will serve as the target cell line for future research.

### Construction of an EMT Model in Gastric Cancer Through Overexpression of Brachyury

**Fig. 2:**
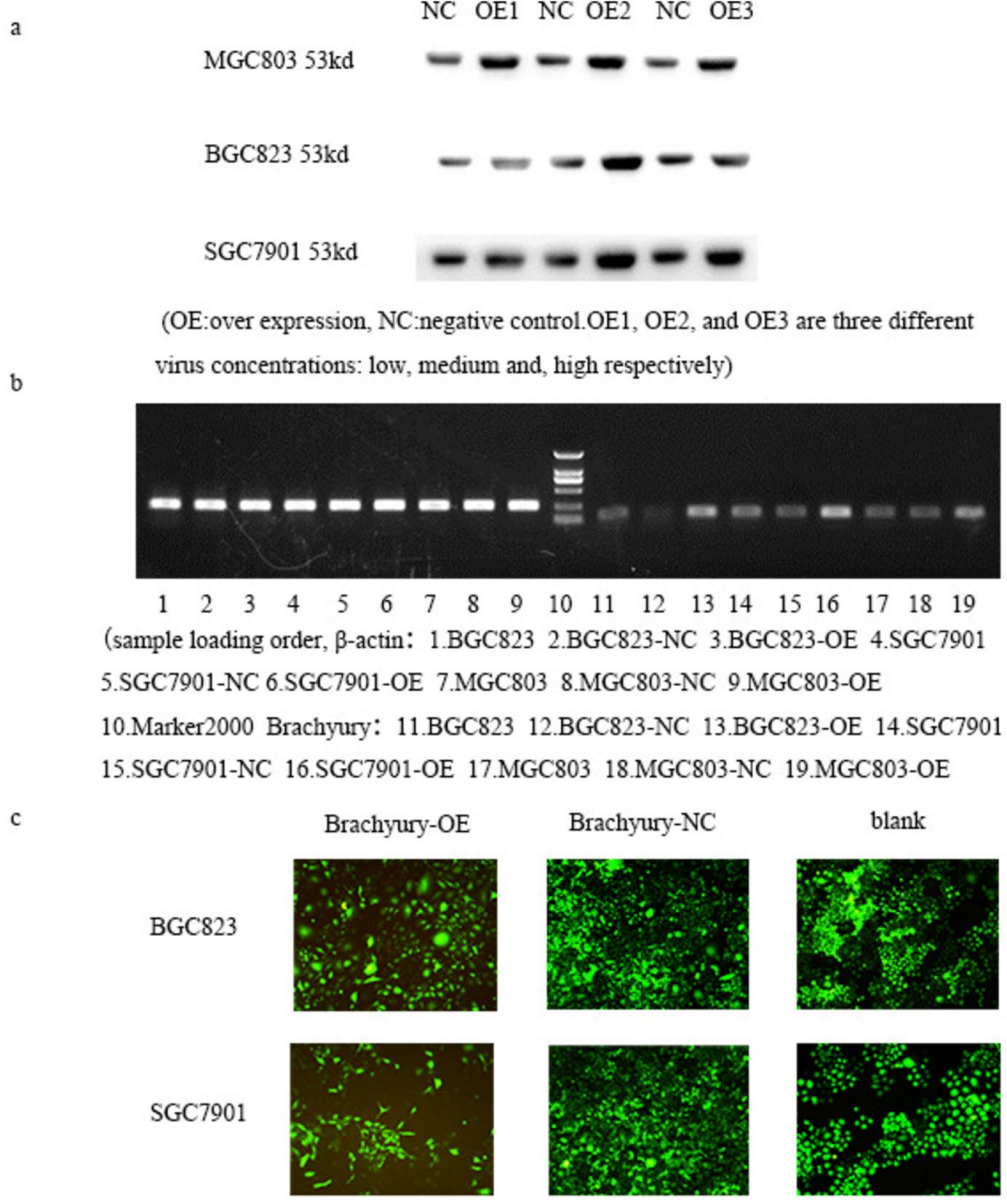
Western Blot (**a**) and qPCR (**b**) analyses of Brachyury protein and gene expression in three cell types 48 h after lentiviral overexpression of Brachyury. **c** Morphological observations of two gastric cancer cell lines overexpressing brachyury, cultured for 2–7 days. After 5 days post-infection with the Brachyury-overexpressing (Brachyury-OE) virus, EMT-related morphological changes were observed in SGC7901 and BGC823 cells. BSD screening, × 10 magnification

We employed lentiviral infection in three distinct cell lines and utilized Western Blot and qPCR techniques to analyze the protein and gene expression levels. Compared to the negative control, we observed a significant increase in Brachyury protein expression (Fig. 2a, b) following viral infection.

We subsequently examined whether the overexpression of Brachyury induced EMT in the gastric cancer cell lines, thereby validating the model’s success. We cultured three gastric cancer cell lines with Brachyury overexpression for 2–7 days to observe changes in cell morphology. Following 48 h of cell culture, we employed immunofluorescence, Western Blot, and qPCR to assess the expression of proteins and genes characteristic of EMT. We also used Transwell assays to evaluate cell invasion and migration capabilities.

We observed that MGC803 cells exhibit a polygonal shape, making it challenging to discern EMT-related morphological changes. However, after 3–4 days of viral infection, noticeable changes in cell morphology were apparent in SGC7901 and BGC823 cells with Brachyury overexpression (Brachyury-OE) compared to the negative control group (Brachyury-NC). EMT-related morphological alterations, including polygonal morphology, became evident. Changes in cell morphology persisted and were still observable after 5–7 days post-infection (Fig. 2c).

### Overexpression of Brachyury Enhances Cell Motility and Induces EMT

**Fig. 3:**
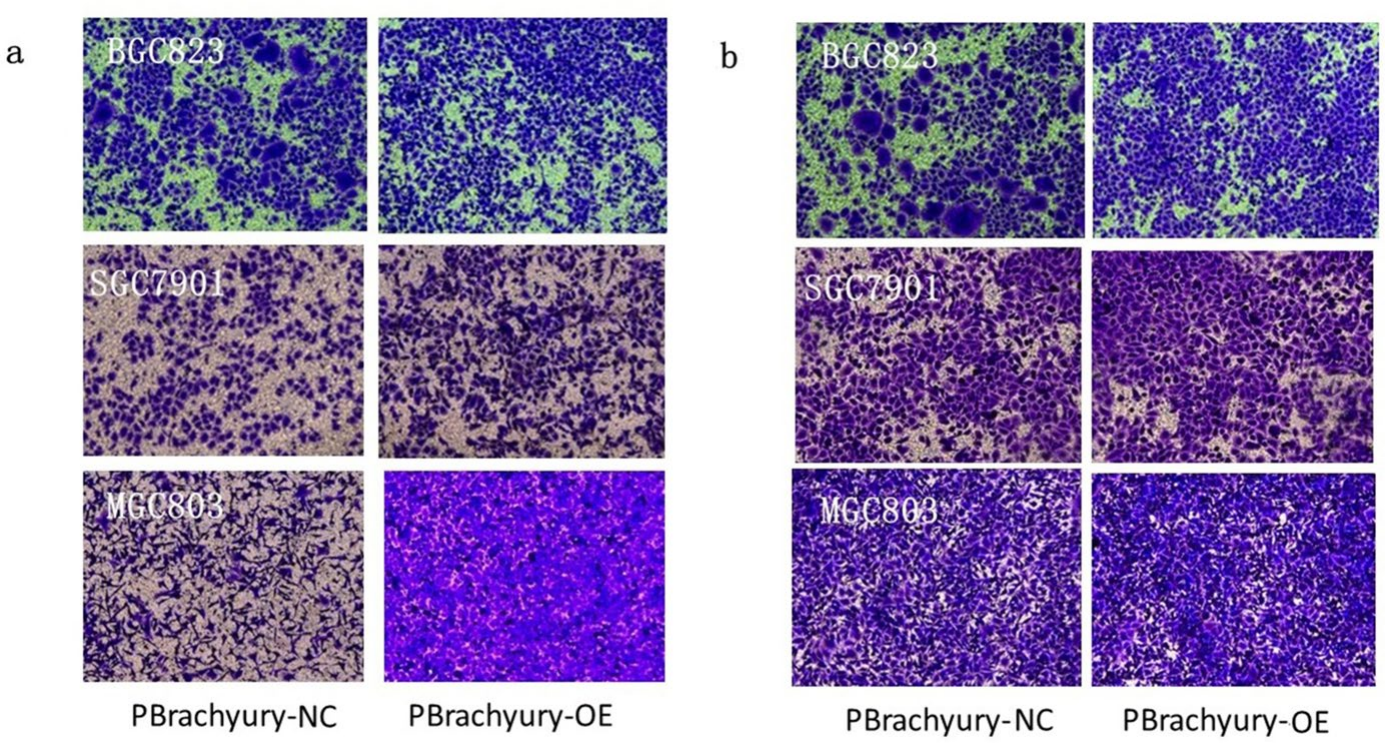
Comparison of migration and invasion capabilities in gastric cancer cells post-brachyury overexpression via lentiviral infection. **a** Transwell Migration and **b** Invasion assays in BGC823, MGC803, and SGC7901 cell lines, 48 h post-Brachyury overexpression via lentiviral infection (× 10 magnification)

Transwell migration assays demonstrated that MGC803 and SGC7901 cells exhibited enhanced migratory capabilities after infection with a Brachyury-overexpressing virus, compared to those infected with a control virus (*P* < 0.05 and *P* < 0.01), while, although BGC823 cells also showed enhanced migration, the difference was not statistically significant (*P* = 0.176) (Fig. 3a). Invasion assays revealed that all three cell types exhibited increased invasive abilities after infection with a Brachyury-overexpressing virus, as compared to control virus-infected cells (*P* < 0.01, *P* < 0.05 and *P* < 0.05) (Fig. 3b).

**Fig. 4:**
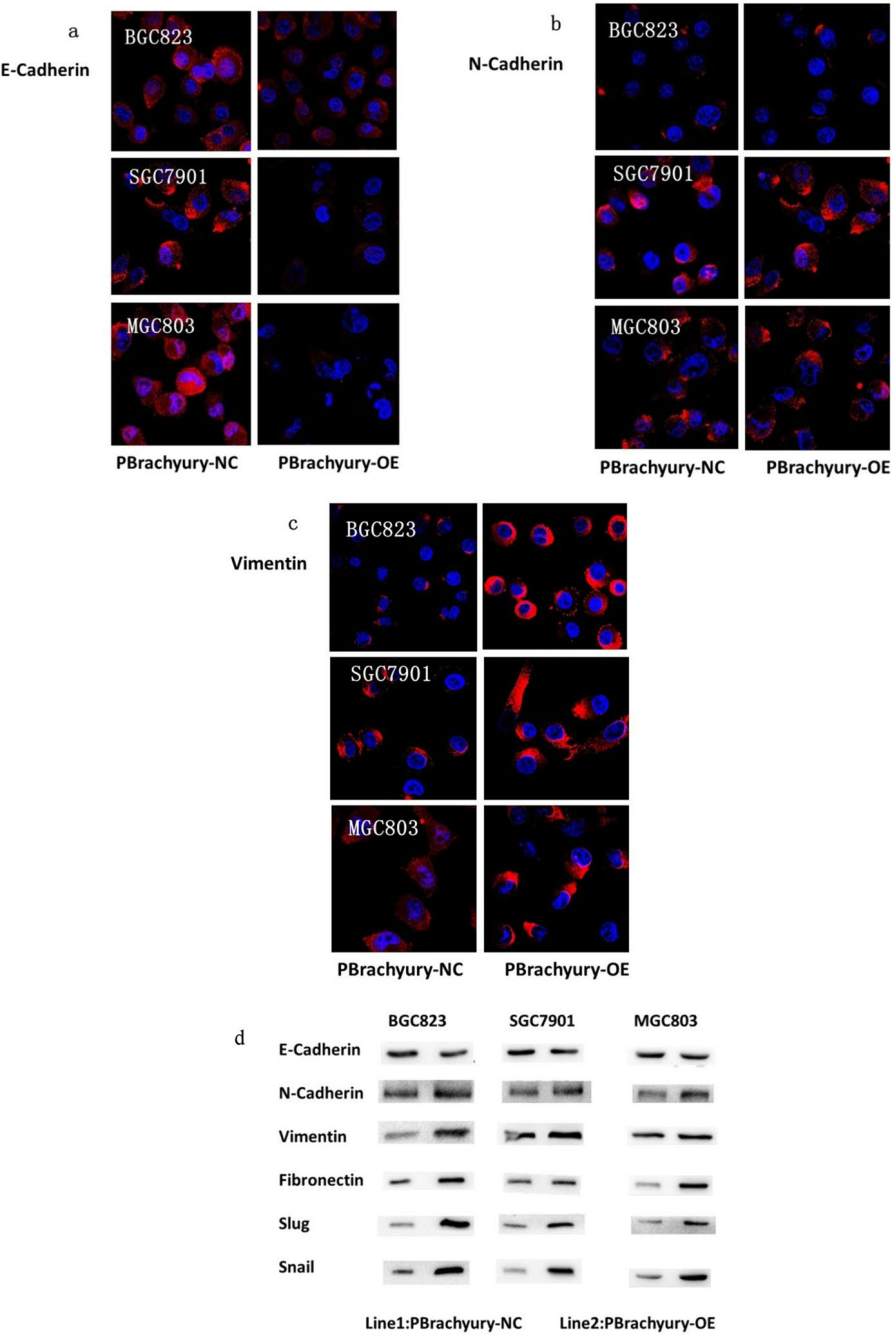
Immunofluorescent analysis of EMT-characteristic protein expression in gastric cancer cells, 48 h post-Brachyury overexpression via lentiviral infection. Nuclei were counterstained with DAPI (blue) (× 63 magnification) (**a**–**c**). EMT-related protein expression detected by western blot in three types of gastric cancer cells (**d**)

Immunofluorescence analysis indicated that E-cadherin expression decreased, while vimentin levels significantly increased in the three cell types infected with the Brachyury-overexpressing virus than in the negative control virus-infected cells (*P* < 0.05). (Fig. 4a–c). Western blot analysis showed significant increases in the expression levels of key proteins such as N-Cadherin (*P* < 0.05), Fibronectin (*P* < 0.05), and the EMT marker protein Snail (*P* < 0.01) of MGC803 cells. In SGC7901 cells, the expression of EMT marker proteins such as Slug (*P* < 0.05) and Snail (*P* < 0.01) was significantly increased. The expression of E-Cadherin in BGC823 cells was significantly reduced (*P* < 0.01), and the expression of Snail was obviously increased (*P* < 0.05) (Fig. 4d).

**Fig. 5:**
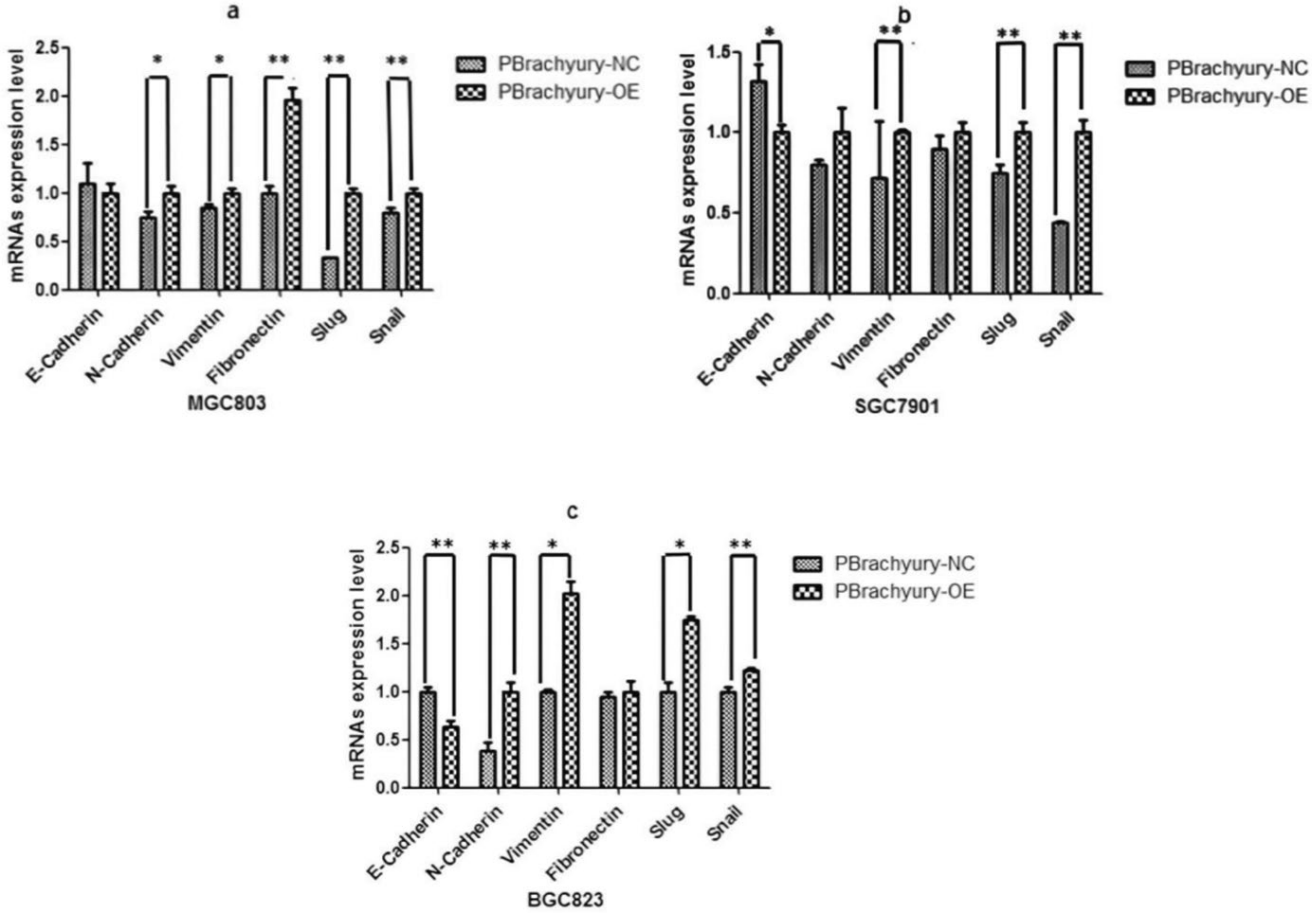
qPCR assays revealed that mRNA expression levels of the characteristic proteins, such as N-Cadherin, Vimentin, Fibronectin, Snail, and Slug, were significantly increased, while the expression level of E-Cadherin was significantly decreased in three gastric cancer cell lines (*P < 0.05 **P < 0.01)

The qPCR analysis revealed that the expression of N-Cadherin (*P* = 0.012), Vimentin (*P* = 0.011), Fibronectin (*P* = 0.001), Slug (P < 0.001), and Snail (*P* = 0.005) in MGC803 cells was significantly increased. Conversely, in SGC7901 cells, E-Cadherin (*P* = 0.017) expression was significantly decreased, while the expression of Vimentin (*P* = 0.001), Slug (*P* = 0.004), and Snail (*P* = 0.006) was significantly elevated. In BGC823 cells, the expression of E-Cadherin (*P* = 0.001) was significantly decreased, while the expression of N-Cadherin (*P* = 0.001), Vimentin (*P* = 0.004), Slug (*P* = 0.002), and Snail (*P* = 0.003) was significantly increased (Fig. 5).

### Expression of IL-8 and Its Receptor Following Brachyury Overexpression in Gastric Cancer Cells

As follow-up studies are planned to intervene with IL-8 and its receptors, clarification of their baseline expression in the EMT model is essential. Using qPCR, we determined that the 2^−△△Ct^ values for SGC7901, MGC803, and BGC823 cells were 1.54 ± 0.10, 1.01 ± 0.14, and 2.01 ± 0.07, respectively (*P* < 0.01; paired comparison *P* < 0.01). BGC823 showed the highest expression, while MGC803 showed the lowest.

ELISA analysis showed that IL-8 expression in the Brachyury-overexpressing groups of SGC7901, BGC823, and MGC803 cells was significantly elevated compared to the Brachyury-negative control group. Specifically, the expression levels increased from 325 ± 13.1 to 904 ± 52.4 pg/ml, from 2630 ± 46.4 to 4298 ± 61.3 pg/ml, and from 458 ± 6.5 to 1235 ± 6.5 pg/ml. Of these, the BGC823 Brachyury-overexpressing group exhibited a notable increase, with all values showing statistical significance (*P* < 0.05).

**Fig. 6:**
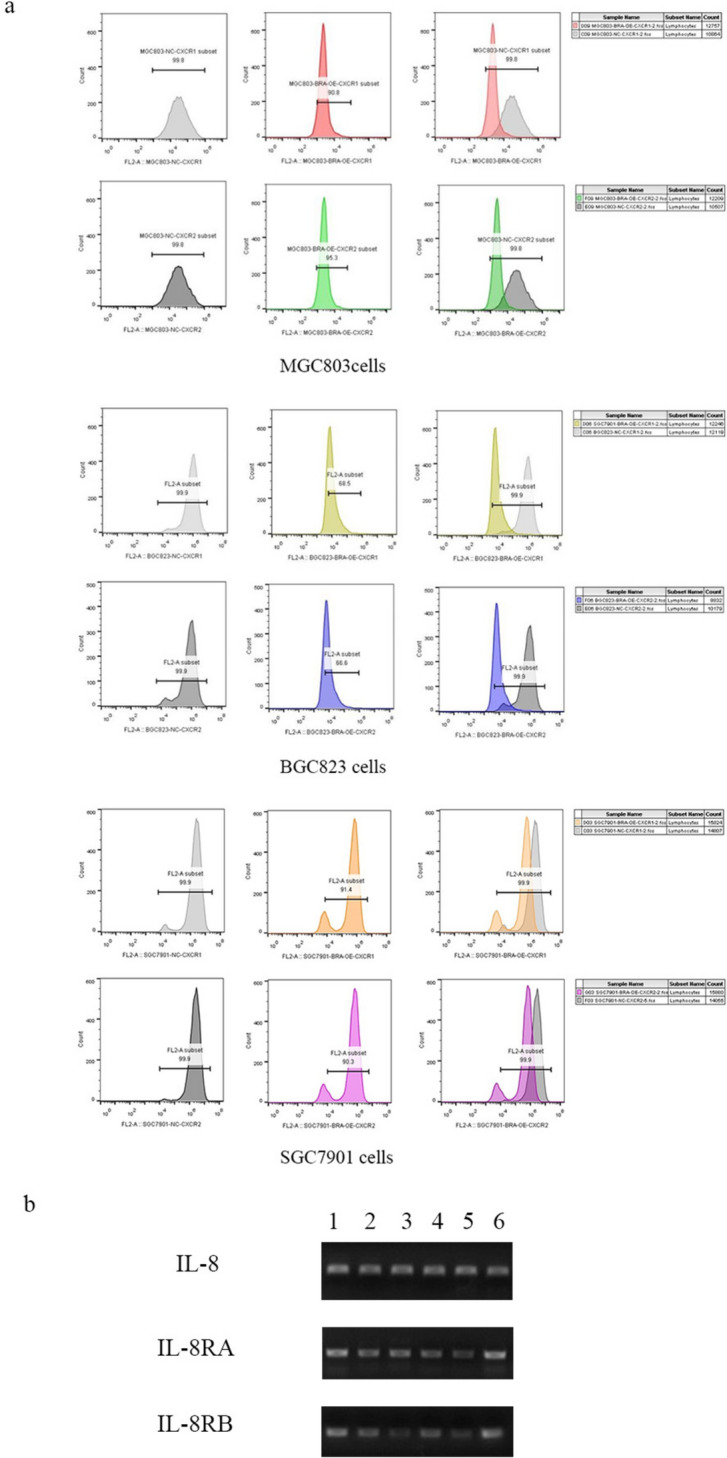
**b** qPCR assays were utilized to detect the expression levels of IL-8, IL-8RA, and IL-8RB mRNA in gastric cancer cells with Brachyury overexpression (1: SGC7901-NC, 2: SGC7901-OE, 3: BGC823-NC, 4: BGC823-OE, 5: MGC803-NC, 6: MGC803-OE)

Using Brachyury-negative cells as the control group, we employed flow cytometry to assess the expression levels of IL-8RA and IL-8RB in gastric cancer cells with Brachyury overexpression. We observed that the expression levels of IL-8RA and IL-8RB were diminished to varying extents, with the reduction in BGC823 cells being particularly pronounced (Fig. 6a).

Interestingly, qPCR analysis revealed discrepancies between mRNA and protein expression levels. Compared to the Brachyury-negative control group, IL-8 mRNA levels increased in SGC7901 cells but decreased in MGC803 and BGC823 cells. Meanwhile, IL-8RA mRNA levels decreased in SGC7901 but increased in BGC823 and MGC803. IL-8RB mRNA levels decreased in both SGC7901 and BGC823, whereas they increased in MGC803 (Fig. 6b).

## Discussion

Recently, growing evidence shows that Brachyury plays a major role in the tumorigenesis and metastasis of various types of human cancers. Shinichiro Shimamatsu et al. found that high expression levels of the Brachyury protein in metastatic carcinoma cells within the intrathoracic lymph nodes are associated with poor prognosis in lung cancer patients (Shimamatsu et al. 2016). Rui Du and colleagues have also shown that the expression of Brachyury correlates with distant metastasis and poor prognosis in hepatocellular carcinoma (HCC) (Du et al. 2014). In this study, we identify the Brachyury genes in gastric cancer cells undergoing EMT in vitro. This characteristic served as a basis for studying EMT and identifying suitable gastric cell lines. We demonstrate that gastric cell lines SGC7901 and BGC823 can be employed to construct an EMT model, as both cell lines exhibit low levels of E-cadherin protein and high levels of Vimentin.

The EMT has also been shown to be an important mechanism contributing to the metastasis and progression of cancer, especially in gastric cancer (Pastushenko and Blanpain 2019; Yao et al. 2021). Recently, Brachyury has been identified as a significant regulator of EMT, modulating the expression of E-cadherin and Vimentin in various cells. Studies have shown that Brachyury acts as a driver of EMT in a diverse range of tumors (Shao et al. 2015; Xu et al. 2015); however, it is unclear whether Brachyury can promote the migration and invasion of gastric cells by regulating the EMT phenotype. In our Transwell assay study, we demonstrated that the overexpression of Brachyury in gastric cancer cells enhanced both migration and invasion. Immunofluorescence analysis revealed that mesenchymal markers were significantly upregulated, while epithelial markers were notably decreased in the Brachyury-overexpressing gastric cancer cells. These results were further validated through Western blotting and qPCR analyses on cultured cells. Consequently, our findings suggest that Brachyury may promote gastric cancer migration and invasion, at least in part, by inducing EMT in gastric cancer cells.

IL-8 (CXCL8), a member of the neutrophil-specific CXC subfamily of chemokines, is a pro-inflammatory chemokine. It is produced by various cell types and serves to recruit leukocytes during infections, tissue injuries, and tumor progression. Previous research has demonstrated that IL-8 is associated with tumor growth, invasion, metastasis, and immune evasion (Lin et al. 2019; Sun et al. 2019; Deng et al. 2020). In our previous study, we demonstrated that IL-8 is associated with the migration and invasion of gastric cancer cells. It also has the capability to inhibit the expression of the EMT-characteristic protein E-cadherin and induce EMT (Ju et al. 2012). Moreover, some studies have identified a positive correlation between IL-8 expression and protein levels of Brachyury in lung tumor tissue (Haro et al. 2013). Our study revealed that in Brazilian gastric cancer cells with overexpressed IL-8, both ELISA and IL-8RA indicated a significant increase in IL-8 expression. Conversely, flow cytometry showed a significant decrease in IL-8RB expression.

## Conclusion

This study has demonstrated that, when compared to existing EMT models, our in vitro model of gastric cancer cells offers a more accurate representation of gastric cancer. Furthermore, we discovered that migration and invasion were enhanced in gastric cancer cells with Brachyury overexpression. Additionally, we observed a significant increase in the expression of IL-8, while the expression of IL-8RA and IL-8RB decreased significantly in the in vitro model. Collectively, the in vitro model offers an opportunity to explore these aspects pertinent to EMT, as they could occur in vivo in gastric cancer, as well as potential drug interactions that may interfere with this process.

## Data Availability

The datasets generated during or analyzed during the current study are available from the corresponding author on reasonable request.
